# Association between intracytoplasmic sperm injection and neurodevelopmental outcomes among offspring

**DOI:** 10.1371/journal.pone.0257268

**Published:** 2021-09-10

**Authors:** Cheng-Wei Wang, Tzu-Hao Chang, Nai-Chen Chuang, Heng-Kien Au, Chi-Huang Chen, Sung-Hui Tseng

**Affiliations:** 1 Division of Reproduction Medicine, Department of Obstetrics and Gynecology, Taipei Medical University Hospital, Taipei, Taiwan; 2 Graduate Institute of Biomedical Informatics, College of Medical Science and Technology, Taipei Medical University, Taipei, Taiwan; 3 Clinical Big Data Research Center, Taipei Medical University Hospital, Taipei, Taiwan; 4 Clinical Data Center, Office of Data Science, Taipei Medical University, Taipei, Taiwan; 5 Department of Obstetrics and Gynecology, School of Medicine, College of Medicine, Taipei Medical University, Taipei, Taiwan; 6 Department of Obstetrics and Gynecology, Taipei Medical University Hospital, Taipei, Taiwan; 7 Department of Physical Medicine and Rehabilitation, Taipei Medical University Hospital, Taipei, Taiwan; 8 Department of Physical Medicine and Rehabilitation, School of Medicine, College of Medicine, Taipei Medical University, Taipei, Taiwan; Universite Clermont Auvergne, FRANCE

## Abstract

**Purpose:**

To compare the risk of neurodevelopmental disorders in children conceived via intracytoplasmic sperm injection (ICSI) and those conceived naturally.

**Materials and methods:**

A population-based cohort study using data retrieved from the Taipei Medical University Research Database (TMURD) from January, 2004 to August, 2016. The data included maternal pregnancy history, perinatal history and developmental follow up of their babies up to 5 years of age. The study included 23885 children, of whom 23148 were naturally conceived and 737 were conceived via ICSI. Neurodevelopmental disorders defined by 21 ICD-9-CM codes.

**Results:**

Of the 23885 children enrolled for analysis, 2778 children were included for further subgrouping analysis after propensity matching to reduce bias from maternal factors. The single-birth group included 1752 naturally conceived (NC) children and 438 ICSI children. The multiple-birth group included 294 NC and 294 ICSI children. The risk of neurodevelopmental disorders was not increased for ICSI children in both groups (single birth: adjusted hazard ratio aHR = 0.70, 95% CI = 0.39–1.27, *p* = 0.243; multiple-birth group aHR = 0.77, 95% CI = 0.43–1.35, *p* = 0.853). In the single-birth group, multivariate analyses showed that male sex (aHR = 1.81, 95% CI = 1.29–2.54, *p* < 0.001), and intensive care unit (ICU) admission (aHR = 3.10, 95% CI = 1.64–5.86, *p* < 0.001) were risk factors for neurodevelopmental disorders. In the multiple-birth group, multivariate analyses demonstrated that ICU admission (aHR = 3.58, 95% CI = 1.82–7.04, *p* < 0.001), was risk factor for neurodevelopmental disorders.

**Conclusion:**

Our study indicated that the use of ICSI does not associated with higher risk of neurodevelopmental disorders in the offspring. But male sex, and ICU admission do have increased risk of neurodevelopmental disorders. However, long term follow up of this cohort on health outcomes in adolescence and adulthood will strengthen the conclusions that ICSI is safe regarding offspring long term outcome.

## Introduction

After the introduction of intracytoplasmic sperm injection (ICSI) in 1992, many infertility centers adopted this procedure as the primary fertilization method because ICSI has proven to be an effective method of fertilization [1]. It bypasses the selection process of natural sperm and is now frequently used in cases of male factor infertility, unexplained infertility, and failed fertilization. The percentage of assisted reproductive technology(ART) programs involving ICSI increased from 47.6% in 2000 to 66% in 2010 and is over 90% in some countries [2]. The increased popularity of ICSI raises questions about the health and development outcomes of ICSI children now and then as many studies offer inclusive results yet [3, 4]. Previous reports of ICSI have focused on neonatal physiological outcomes such as body weight, height, blood pressure, and congenital anomalies [5, 6]. Other studies have reported inconsistent results for neurodevelopmental sequelae in children conceived using ART including ICSI [7–10]. A recent systematic review reported inconclusive findings about the impact of ICSI on intelligence [11]. ICSI related technique, and process during critical window of epigenetic reprogramming has been one of the suggested mechanisms involved in the pathogenesis in a number of neurodevelopmental disorders in the offsprings [12]. These results suggest that the developmental aspects of ICSI-conceived children need more definite evidence.

In this study, our objective is to examine the association of ICSI by compare the risk of neurodevelopmental disorders in children conceived via ICSI and those conceived naturally based on Taipei Medical University Research Database (TMURD) which include the documentation of standardized health care process for ICSI, antenatal care, and development evaluation from 3 affiliated hospitals of Taipei Medical University.

## Materials and methods

Data for the present study were retrieved Taipei Medical University Clinical Research Database (TMUCRD), which contains the electronic health records of more than 3 million patients from three affiliated teaching hospitals: Taipei Medical University Hospital (TMUH), Wan Fang Hospital (WFH), and Shuang Ho Hospital (SHH); patient profiles during the period January, 2004 to August, 2016 were used for patient enrollment. ICSI is done step by step, and each step has a billing code. Antenatal care of naturally conceived pregnancy also has well established timelines and billing codes in Taiwan. According to Children and Youth Welfare Law of Taiwan, children with developmental delay/disorders are those who have been evaluated by health ministry accredited multidisciplinary medical teams. Then the children will be eligible to receive early intervention services. Standardized health care process reduced risk of misinterpretations and allows aggregate of data from different hospitals. All three hospitals have health ministry accredited multidisciplinary medical teams to perform developmental evaluation and assessment. This study was approved by the Institutional Review Board of Taipei Medical University (TMU-JIRB-201706049). The institutional research board of the TMU-JIRB approved the study and waived the requirement for informed consent and the further need of guidelines.

Inclusion criteria for the study included pregnant women who underwent a prenatal examination and gave birth between January, 2004 and August, 2016. Exclusion criteria included pregnant women who were under 20 years old, missing birth date of the newborn, misreported sex of pregnant woman, and non-ICSI patients. The included pregnant women were divided into an ICSI group and a natural-conception group. The ICSI group was defined as patients with ICSI and embryo transfer at least 300 days before childbirth. The non-ICSI group was defined as patients with embryo transfer at least 300 days before childbirth but without receiving ICSI. The natural-conception group was defined as patients without embryo transfer at least 300 days before childbirth.

Demographic data included maternal age, type of birth, maternal hospitalization history, maternal medication use, sex of newborn, gestation weeks, birth weight, neonatal intensive care unit(ICU) admission, and neonatal condition (ICD-9-CM 760–779). In this study, children with neurodevelopmental disorders were defined as those who had any of the following ICD-9-CM codes at least twice in EHR during the study period: brain hypoxia (ICD-9-CM 348), infantile cerebral palsy (ICD-9-CM 343), autism (ICD-9-CM 299), Attention-deficit/hyperactivity disorder (ADHD) (ICD-9-CM 314), intellectual disability (ICD-9-CM 317–319), developmental disabilities (ICD-9-CM 315), other speech disturbance (ICD-9-CM 7845), voice disturbance (ICD-9-CM 7844), aphasia (ICD-9-CM 7813), abnormality of gait (ICD-9-CM 7812), epilepsy (ICD-9-CM 345), blindness or low vision (ICD-9-CM 369), hearing loss (ICD-9-CM 389), anencephaly and similar anomalies (ICD-9-CM 740), chromosomal anomalies (ICD-9-CM 758), lack of expected normal physiological development in childhood (ICD-9-CM 7834), other developmental disorder (ICD-9-CM 783.9), other symbolic dysfunction (ICD-9-CM 7846), and special symptoms or syndromes not elsewhere classified (ICD-9-CM 307). Neonatal conditions identified by any of ICD-9 codes 760–779 in the perinatal period.

Descriptive statistics were used to summarize the demographic data. Continuous variables are presented as mean and standard deviation (SD), categorical variables are presented as the number of subjects and percentage (%). Propensity score matching (PSM) was used to reduce bias from maternal factors, including maternal age, maternal hospitalization history, and maternal medication use. Before PSM, Student’s *t* test was used to assess maternal age, gestation weeks, and birth weight; and the chi-squared test or Fisher’s exact test were used for type of birth, maternal hospitalization history, maternal medication use, sex of newborn, ICU admission, and neonatal condition. Subgroup analysis was used to determine the risk factors for developmental disabilities in the single-birth and multiple-birth groups. After PSM, the differences between matched pairs were evaluated using the signed rank test for continuous data and McNemar’s test for binary data. Cox proportional hazards regression was used to determine the risk factors for developmental disabilities by including all the candidate variables in the model. A two-sided statistical test at 5% significance was used. All of the analyses were performed using SAS Enterprise Guide software version 7.11 (SAS Institute, Cary, NC, USA).

## Results

**Fig 1:**
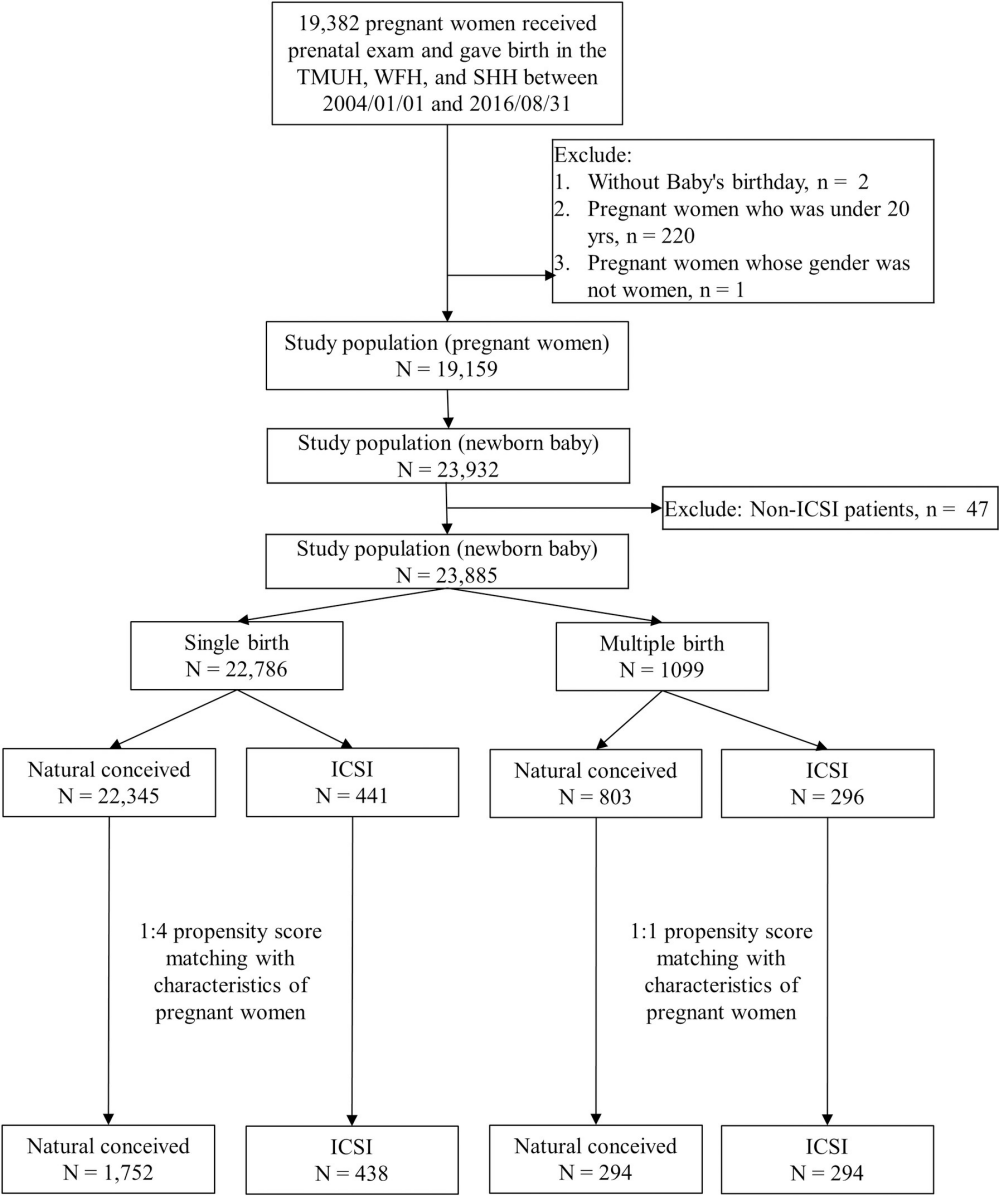
Inclusion and exclusion criteria flow diagram.

We performed a chart review for 19,382 pregnant women underwent a prenatal examination and gave birth in TMUH, WFH, and SHH during the study period. After removing the data for individuals who met the exclusion criteria, 23,885 newborns were enrolled in the study, including 22,786 singleton babies and 1,099 multiple-birth babies (Fig 1). The mean follow-up time was 3.3 ± 2.3 years. The baseline characteristics of the pregnant women and their newborns included in the study are shown in Table 1. The maternal age was significantly higher in the ICSI group than in the natural-conception group (36.5 ± 3.7 years vs 32.0 ± 4.6 years, *p* < 0.001). In contrast, birth weeks and birth weights were significantly lower in the ICSI group than in the natural-conception group (36.6 ± 3.0 weeks vs 38.5 ± 1.8 weeks, *p* < 0.001; 2639.8 ± 679.0 g vs 3092.5 ± 477.8 g, *p* < 0.001, respectively). The proportions of multiple births, maternal medication use, and ICU admissions were significantly higher in the ICSI group than in the natural-conception group (all *p* ≤ 0.001). In contrast, the proportion of male babies was significantly lower in the ICSI group than in the natural-conception group (47.6% vs 51.7%, *p* = 0.033).

**Table 1:** Original characteristics of pregnant women and newborn baby in the study population.

	Total	Natural conceived	ICSI	
	(n = 23,885)	(n = 23,148)	(n = 737)	
	N (%)	N (%)	N (%)	p-value
Maternal age (yrs)	32.2 ± 4.6	32.0 ± 4.6	36.5 ± 3.7	**< 0.001**
Type of birth				**< 0.001**
Single birth	22786 (95.4)	22345 (96.5)	441 (59.8)	
Multiple birth	1099 (4.6)	803 (3.5)	296 (40.2)	
Hospitalization of pregnant women	840 (3.5)	823 (3.6)	17 (2.3)	0.083
Medication of pregnant women	381 (1.6)	354 (1.5)	27 (3.7)	**< 0.001**
Gender of newborn baby, male	12311 (51.5)	11960 (51.7)	351 (47.6)	**0.033**
Birth weeks, n = 23,533	38.4 ± 1.9	38.5 ± 1.8	36.6 ± 3.0	**< 0.001**
Birth weights, g, n = 23,569	3077.5 ± 491.6	3092.5 ± 477.8	2639.8 ± 679.0	**< 0.001**
ICU	799 (3.3)	694 (3.0)	105 (14.2)	**< 0.001**
Neonatal condition	15413 (64.5)	14918 (64.4)	495 (67.2)	0.137

Data period: 2004/01/01 ~ 2016/08/31.

ICSI, IntraCytoplasmic Sperm Injection; ICU, Intensive Care Unit.

Continuous variables are presented as mean and standard deviation (SD), categorical variables are presented as number of subjects and percentage (%).

Student’s t test was used for maternal age, baby birth weeks, and baby birth weights; and Chi-squared test or Fisher’s exact test were used for abortion of pregnant women, type of birth, hospitalization of pregnant women, medication of pregnant women, ICU, and neonatal condition.

Boldface is presented as statistically significant difference (p-value < 0.05).

Subgroup analysis was used to determine the risk factors for neurodevelopmental disorders separately in single-birth and multiple-birth groups. In the single-birth group, 22,345 naturally conceived babies and 441 ICSI babies were included (Fig 1), and the maternal age was significantly higher for the ICSI babies than for the naturally conceived babies (36.9 ± 3.6 years vs 32.0 ± 4.5 years, *p* < 0.001). In contrast, gestation weeks (38.0 ± 2.3 weeks vs 38.6 ± 1.7 weeks, *p* < 0.001) and birth weight (2984.4 ± 553.2 g vs 3119.5 ± 455.1 g, *p* < 0.001) were still significantly lower for the ICSI babies than for the naturally conceived babies. The proportions of maternal medication use (4.8% vs 1.5%, *p* < 0.001) and neonatal ICU admission (6.8% vs 2.6%, *p* < 0.001) were significantly higher in the ICSI group than in the natural-conception group. In contrast, the proportion of male babies was significantly lower in the ICSI group than in the natural-conception group (45.8% vs 51.8%, *p* = 0.014) (Table 2). To reduce selection bias, 1:4 PSM was used to match the characteristics of the pregnant women, such as maternal age, maternal hospitalization history, and maternal medication use, in the single-birth group. After PSM, the final analyses included 1,752 naturally conceived babies and 438 ICSI babies (Fig 1). Table 3 shows that none of the characteristics of the pregnant women differed significantly between the natural-conception and ICSI groups. The trends for the other variables were similar to those before matching.

**Table 2:** Characteristics of pregnant women and newborns for patients with single and multiple births.

	Single birth				Multiple births			
	Total	Naturally conceived	ICSI		Total	Naturally conceived	ICSI	
	(n = 22,786)	(n = 22,345)	(n = 441)		(n = 1,009)	(n = 803)	(n = 296)	
	N (%)	N (%)	N (%)	p-value	N (%)	N (%)	N (%)	p-value
Maternal age (years)	32.1 ± 4.6	32.0 ± 4.5	36.9 ± 3.6	**<0.001**	34.1 ± 4.3	33.4 ± 4.4	35.9 ± 3.7	**<0.001**
Maternal hospitalization history	768 (3.4)	759 (3.4)	9 (2.0)	0.141	72 (6.6)	64 (8.0)	8 (2.7)	**0.001**
Maternal medication use	363 (1.6)	342 (1.5)	21 (4.8)	**<0.001**	18 (1.6)	12 (1.5)	6 (2.0)	0.593
Sex of newborn, male	11773 (51.7)	11571 (51.8)	202 (45.8)	**0.014**	538 (49.0)	389 (48.4)	149 (50.3)	0.587
Gestation weeks	38.6 ± 1.7	38.6 ± 1.7	38.0 ± 2.3	**<0.001**	35.4 ± 2.5	35.7 ± 2.4	34.5 ± 2.7	**<0.001**
Birth weight	3116.8 ± 457.5)	3119.5 ± 455.1	2984.4 ± 553.2	**<0.001**	2279.3 ± 496.6	2336.9 ± 483.0	2124.6 ± 500.1	**<0.001**
ICU admission	620 (2.7)	590 (2.6)	30 (6.8)	**<0.001**	179 (16.3)	104 (13.0)	75 (25.3)	**<0.001**
Neonatal condition	14621 (64.2)	14353 (64.2)	268 (60.8)	0.133	792 (72.1)	565 (70.4)	227 (76.7)	**0.041**

Data period: 2004/01/01-2016/08/31.

ICSI, Intracytoplasmic sperm injection; ICU, intensive care unit.

Continuous variables are presented as mean and standard deviation (SD); categorical variables are presented as the number of subjects and percentage (%).

Student’s t test was used for maternal age, baby gestation weeks, and baby birth weight; chi-squared test or Fisher’s exact test was used for maternal hospitalization history, maternal medication use, ICU admission, and neonatal condition.

Boldface indicates a significant difference (*p* < 0.05).

**Table 3:** Matched characteristics of pregnant women and newborns for patients with single and multiple births.

	Single birth				Multiple births			
	Total	Naturally conceived*	ICSI*		Total	Naturally conceived*	ICSI*	
	(n = 2,190)	(n = 1,752)	(n = 438)		(n = 588)	(n = 294)	(n = 294)	
	N (%)	N (%)	N (%)	p-value	N (%)	N (%)	N (%)	p-value
Maternal age (years)	36.8 ± 3.5	36.8 ± 3.5	36.8 ± 3.5	0.969	35.8 ± 3.6	35.8 ± 3.7	35.8 ± 3.6	0.929
Maternal hospitalization history	34 (1.6)	25 (1.4)	9 (2.1)	0.385	16 (2.7)	8 (2.7)	8 (2.7)	1.000
Maternal medication use	97 (4.4)	78 (4.5)	19 (4.3)	0.917	14 (2.4)	8 (2.7)	6 (2.0)	0.788
Sex of newborn, male	1145 (52.3)	945 (53.9)	200 (45.7)	**0.002**	293 (49.8)	145 (49.3)	148 (50.3)	0.869
Gestation weeks	38.3 ± 2.1	38.4 ± 2.0	38.0 ± 2.4	**0.002**	35.2 ± 2.5	35.8 ± 2.1	34.5 ± 2.7	**<0.001**
Birth weight, g	3096.5 ± 516.8	3125.3 ± 503.2	2982.5 ± 553.6	**<0.001**	2231.0 ± 495.3	2341.5 ± 466.6	2121.3 ± 499.3	**<0.001**
ICU admission	95 (4.3)	65 (3.7)	30 (6.8)	**0.006**	116 (19.7)	41 (13.9)	75 (25.5)	**0.001**
Neonatal condition	1375 (62.8)	1110 (63.4)	265 (60.5)	0.270	415 (70.6)	189 (64.3)	226 (76.9)	**0.001**

Data period: 2004/01/01-2016/08/31.

ICSI, Intracytoplasmic sperm injection; ICU, intensive care unit.

Continuous variables are presented as mean and standard deviation (SD); categorical variables are presented as the number of subjects and percentage (%).

* The results were performed by 1:4/ 1:1 propensity score matching on single/ multiple births patients, matching variables included maternal age, maternal hospitalization history, and maternal medication use. The differences between matched pairs were evaluated using the signed rank test for continuous data and the McNemar’s test for binary data.

Boldface indicates a significant difference (*p* < 0.05).

The multiple-birth group included 803 naturally conceived babies and 296 ICSI babies (Fig 1). The trends for all of the variables in the multiple-birth group were similar to those for the single-birth group, except for maternal hospitalization history, maternal medication use, the sex of the newborn, and neonatal condition (Table 2). The proportion of mothers with a history of hospitalization was significantly lower in the ICSI group than in the natural-conception group (2.7% vs 8.0%, *p =* 0.001). Rates of maternal medication use and the sex of the newborn baby did not differ significantly between the two groups. Rate of neonatal condition was significantly higher in the ICSI group than in the natural-conception group (76.7% vs 70.4%, Table 2). After 1:1 PSM was used to match the characteristics of the pregnant women in the multiple-birth group, 294 naturally conceived babies and 294 ICSI babies were included (Fig 1). Table 3 shows that the results for gestation weeks, birth weight, ICU admissions, and the neonatal condition were similar to those before matching.

**Fig 2:**
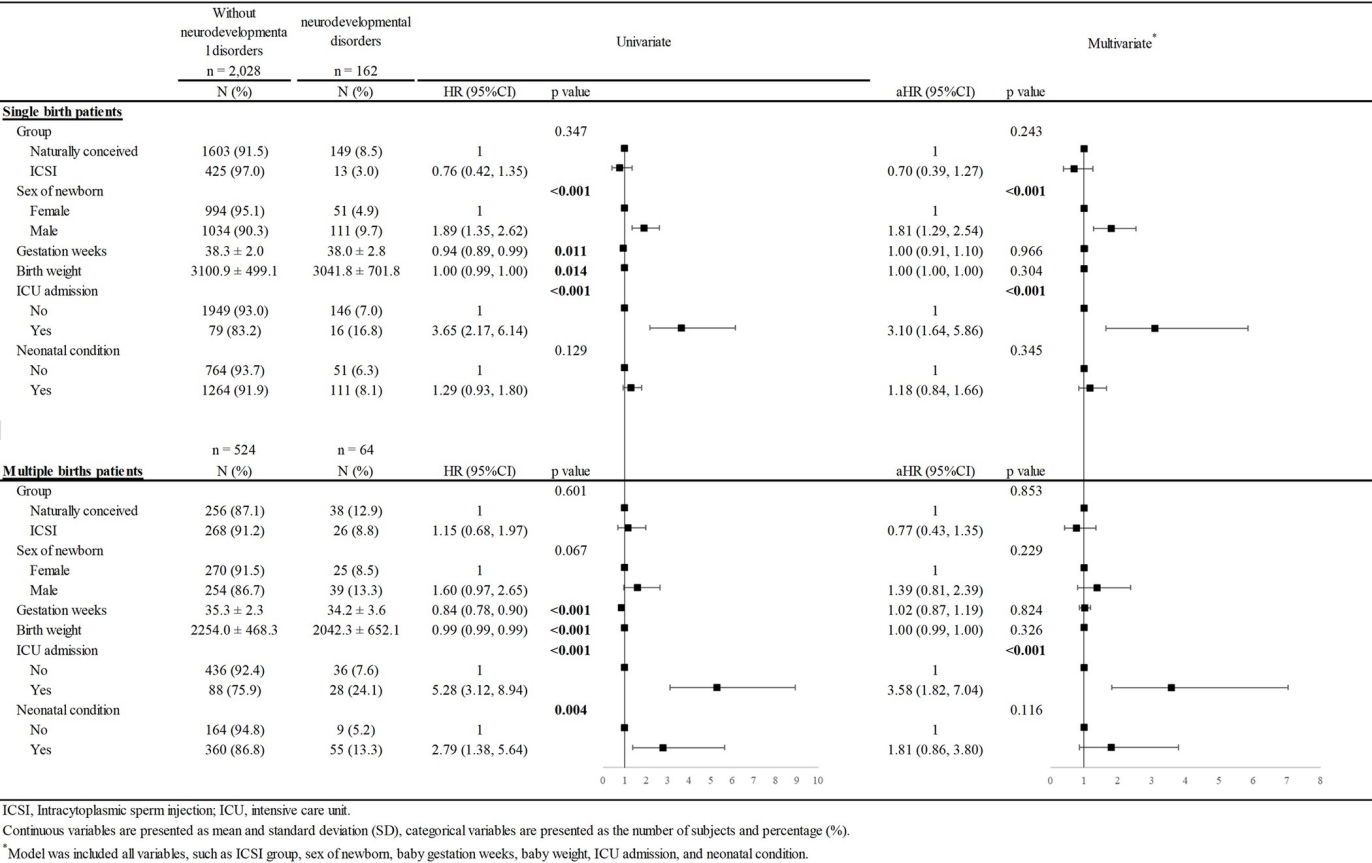
Univariate and multivariate cox regression analysis of neurodevelopmental disorders in subgroup analysis.

The outcome of the Cox proportional hazards regression analysis is summarized in Fig 2. In the single-birth group, the univariate and multivariate analyses both demonstrated that male sex (hazard ratio [HR] = 1.89, 95% confidence interval [CI] = 1.35–2.62, *p* < 0.001; adjusted HR [aHR] = 1.81, 95% CI = 1.29–2.54, *p* < 0.001) and ICU admission (HR = 3.65, 95% CI = 2.17–6.14, *p* < 0.001; aHR = 3.10, 95% CI = 1.64–5.86, *p* < 0.001) were risk factors for neurodevelopmental disorders. However, although gestation weeks was associated with a significantly lower risk in the univariate model, the significance disappeared in the multivariate analysis (HR = 0.94, 95% CI = 0.89–0.99, *p* = 0.011; aHR = 1.00, 95% CI = 0.91–1.10, *p* = 0.966). In the multiple-birth group, the univariate and multivariate analyses both indicated that ICU admission (HR = 5.28, 95% CI = 3.12–8.94, *p* < 0.001; aHR = 3.58, 95% CI = 1.82–7.04, *p* < 0.001) was risk factors for neurodevelopmental disorders. However, gestation weeks and birth weight were associated with a significantly lower risk in univariate analysis but not in multivariate analysis. The neonatal condition was associated with a significantly higher risk in univariate analysis but not in multivariate analysis (HR = 2.79, 95% CI = 1.38–5.64, *p* = 0.004; aHR = 1.81, 95% CI = 0.86–3.80, *p* = 0.116). After adjusting for all variables, the ICSI group does not increase the risk of neurodevelopmental disorders compared with the natural-conception group in both single- and multiple-birth groups (aHR = 0.70, 95% CI = 0.39–1.27, *p* = 0.243; aHR = 0.77, 95% CI = 0.43–1.35, *p* = 0.853, respectively).

## Discussion

In this cohort study, we compared neurodevelopmental consequences of the children born after ICSI with those conceived naturally by analyzing both maternal and offspring factors. The ICSI mothers were older (36.5 years vs 32.2 years, *p* < 0.001) and had a higher rate of medication use (3.7% vs 1.6%, *p* = 0.001) (Tables 1 and 2). ICSI had significantly higher rates of multiple live births, preterm birth, low birth weight, and ICU admission. The results are in line with the previous studies that women enter infertility programs at an older age, and ART procedures generally result in a higher rate of multiple pregnancies [13]. In addition, ICSI mothers may use more medication than natural-conception mothers for two reasons: first, ART requires luteal support up to at least 8 weeks of gestation, and second, obstetrical complications increase with increasing maternal age and multiple pregnancies, as seen in the ICSI group.

In this study, neonatal conditions are identified by any of ICD-9 codes 760–779 in the perinatal period, which include conditions not severe. This may be the reason that in multivariate analysis, the neonatal condition only showed trend of increased association with neurodevelopmental disorders (aHR = 1.81, 95% CI = 0.86–3.80, p = 0.116). However, ICU admission appears to be a significant risk factor for neurodevelopmental disorders (HR = 3.65, 95% CI = 2.17–6.14, p < 0.001; aHR = 3.10, 95% CI = 1.64–5.86, p < 0.001), after we matched for maternal conditions and performed univariate and multivariate regression analysis. The result suggests that as the neonatal ICU admission means that the baby has more serious etiologic indications for admission which impact the development of the babies born after ICSI or spontaneous conception [14]. Therefore, early and regular developmental surveillance and assessment are warranted in this group of children.

Our results are in line with other studies that ICSI and naturally conceived children have comparable risks of developmental disabilities [15, 16]. In genomic era, more studies have reported de novo mutations as the cause of developmental disorders such as autism and intellectual disabilities [17, 18]. Germline de novo mutation has been associated with increased paternal age at conception [19]. In ICSI, a single sperm is selected and injected directly into an oocyte to complete fertilization, while natural conception involves the natural selection of competent sperm. The procedure probably reduced the so called paternal age effect.

Many factors undermine poor child development [20]. The strength of this study is that we investigated the causality of ICSI offspring development outcome by matching obstetric factors and neonatal factors. But one of the limitations of this study is that we did not include paternal age factor in our analysis. Although in the present study we matched for some maternal factors, other maternal factors, such as preeclampsia [21], uterine myoma [22], adenomyosis [23], and socioeconomic factors, could have contributed to preterm labor and ICU admission [24]. To ensure the accuracy of the diagnosis of neurodevelopmental disorders, we used patients for whom the same disease code appeared more than twice in the database. Patients with a single coded visit were not included, which may have excluded some part of a potential diseased population. Therefore, the sample size of the developmental disability group in this study was relatively small compared with the normal group. In addition, because the average follow-up period in this study was 3 to 5 years, the development outcomes after 5 years old are unknown.

In conclusion, this study makes an important contribution to the body of evidence concerning the association of neurodevelopmental disorders with ICSI. The findings suggest that ICSI is not associated with increased risk of neurodevelopmental disorders, while male sex, and ICU admission increase the risk of neurodevelopmental disorders in both single and multiple births. However, studies with larger sample sizes and longer follow up are needed to confirm these findings.
